# A prognostic model based on cell-cycle control predicts outcome of breast cancer patients

**DOI:** 10.1186/s12885-020-07045-3

**Published:** 2020-06-16

**Authors:** Heli Repo, Eliisa Löyttyniemi, Samu Kurki, Lila Kallio, Teijo Kuopio, Kati Talvinen, Pauliina Kronqvist

**Affiliations:** 1 Institute of Biomedicine, University of Turku, Turku, Finland; 2 Central Hospital of Central Finland, Jyväskylä, Finland; 3 Department of Biostatistics, University of Turku, Turku, Finland; 4 Turku University Hospital, Turku, Finland; 5 Department of Pathology, University of Turku, Kiinamyllynkatu 10/MedD5A, 20500 Turku, Finland

**Keywords:** Breast cancer, Prognosis, Proliferation, Cell cycle, Securin, Separase

## Abstract

**Background:**

A prognostic model combining biomarkers of metaphase-anaphase transition of the cell cycle was developed for invasive breast cancer. The prognostic value and clinical applicability of the model was evaluated in comparison with the routine prognosticators of invasive breast carcinoma.

**Methods:**

The study comprised 1135 breast cancer patients with complete clinical data and up to 22-year follow-up. Regulators of metaphase-anaphase transition were detected immunohistochemically and the biomarkers with the strongest prognostic impacts were combined into a prognostic model. The prognostic value of the model was tested and evaluated in separate patient materials originating from two Finnish breast cancer centers.

**Results:**

The designed model comprising immunoexpressions of Securin, Separase and Cdk1 identified 8.4-fold increased risk of breast cancer mortality (*p* < 0.0001). A survival difference exceeding 15 years was observed between the majority (> 75%) of patients resulting with favorable as opposed to unfavorable outcome of the model. Along with nodal status, the model showed independent prognostic impact for all breast carcinomas and for subgroups of luminal, N+ and N- disease.

**Conclusions:**

The impact of the proposed prognostic model in predicting breast cancer survival was comparable to nodal status. However, the model provided additional information in N- breast carcinoma in identifying patients with aggressive course of disease, potentially in need of adjuvant treatments. Concerning N+, in turn, the model could provide evidence for withholding chemotherapy from patients with favorable outcome.

## Background

Cell proliferation, hormone-regulation and HER2 amplification are considered the main biological processes driving breast cancer progression. Although proliferation has been shown a valid prognosticator in all subtypes, particularly triple-negative breast carcinoma (TNBC) has been characterized by high expression of proliferation-related genes [1, 2]. The prognostic value of proliferation is acknowledged in the clinical pathology practice as part of the traditional histological grading as well as in intrinsic classification and in modern personalized signatures retrieved from microarray-based expression-profiling [3–5]. However, the impact of deregulated proliferation is still not accurately reflected in the routine clinical parameters and pathological markers applied to treatment decisions of breast cancer patients.

Genetic integrity of the dividing cell is ensured by complex and intricately monitored cellular events at the metaphase-anaphase transition of the cell cycle [6]. Dysfunction of these regulators can lead into missed sister chromatid separation, chromosomal instability and aneuploidy. Premature sister chromatid separation is prevented by the highly controlled sequential activation and inactivation of a cascade of regulatory proteins, particularly Cdc20 (cell division cycle 20), Cohesin, Separase (Extra Spindle Pole Bodies Like protein 1, ESPL1), Securin (Pituitary tumor-transforming gene 1, Pttg1), Pttg1IP (Pituitary tumor-transforming gene 1 interacting protein, also Pituitary tumor-transforming gene 1 binding factor, PBF), Cdk1 (Cyclin-Dependent Kinase protein 1) and CyclinB1 (G2/mitotic-specific cyclin-B1). In more detail, correct segregation of the chromosomes is triggered at the Spindle Assembly Checkpoint (SAC) controlled by Cdc20 activating Anaphase-Promoting Complex / Cyclosome (APC/C) to create the APC/C^Cdc20^ complex. Throughout metaphase, contact between the chromatids is maintained by rings of Cohesin. At the initiation of anaphase, Cohesin is removed triggered by Separase and activated by degradation of Securin and/or the Cdk1/CyclinB1 complex, leading to separation of the sister chromatids [7, 8].

In our previous research, *PTTG1,* the gene of human Securin, was detected with the most significant expression difference between human breast cancer and normal breast glandular tissue on basis of a cDNA microarray analysis involving 4000 cancer related genes [9]. In addition to Securin, also several other regulators of metaphase-anaphase transition have been shown with independent prognostic impacts in breast cancer [10–18]. In the present study, we introduce on basis of a total of 1135 patients with up to 22-year follow-up, a clinically applicable combination of biomarkers of metaphase-anaphase transition leading to optimal detection of aggressive course of disease and cancer mortality in invasive breast cancer.

## Methods

### Patient materials

The study comprises patients diagnosed and treated with unilateral invasive breast carcinoma in two different institutions (Table 1). The first cohort (I) (*n* = 781) originated from the Central Hospital of Central Finland, Jyväskylä, Finland, from years 1987–1997 resulting in maximum follow-up time of 22.7 years. The second cohort (II) (*n* = 354) was collected from Turku University Hospital and Auria biobank, Turku, Finland. This cohort was classified into intrinsic subgroups comprising 208 patients with luminal and 148 patients with triple-negative breast carcinomas diagnosed and treated during 2005–2015 resulting in maximum follow-up times of 14 and 17.8 years, respectively.

**Table 1:** Summary of patient cohorts with clinico-patohologic characteristics

	Cohort I	Cohort II	
	All subtypes (*n* = 781)	Luminal (*n* = 208)	TNBC (*n* = 146)
Mean age at diagnosis (range) (years)	61 (28–95)	62 (42–76)	60 (39–78)
Axillary lymph node positive (%)	44.8	22.8	32.1
Mean tumor size (range) (cm)	2.3 (0.2–16.0)	1.9 (0.4–7.0)	2.5 (0.2–18.0)
Histological type (%)			
Infiltrating ductal NOS	75. 4	72.3	100
Special type	24.6	27.2	0
Intrinsic subtype (%)			
Luminal	67.6	100	–
Her2-amplified	18.6	–	–
Triple-negative	13.8	–	100
Histological grade (%)			
Low (1-2)	79.6	81.6	0
High (3)	20.4	18.4	100
Median follow-up time (max) (years)	12.4 (22.7)	11.8 (14.0)	6.9 (17.8)
Dead of breast cancer (%)	30.7	10.7	22.6

For all patients, the biomarkers of the metaphase-anaphase transition were immunohistochemically (IHC) detected, and the routine clinico-pathological prognostic features of breast cancer were collected. For both cohorts of breast cancer patients, a prognostic model was assembled based on the optimal combination of biomarkers of the metaphase-anaphase transition. The prognostic value of the models was evaluated in comparison with the clinically applied routine prognosticators of breast cancer.

All patients were treated with surgical resection or mastectomy with axillary evacuation, radiation and/or adjuvant treatment with anti-estrogenic or cytostatic drugs depending on the patients’ age, hormone receptor and lymph node (N) status according to the international guidelines for breast cancer treatment at the time of diagnosis [19]. No pre-operative adjuvant treatments were administered. Complete clinical data was collected from pathology reports and patient files and registered applying the criteria presented by WHO [20] and St. Gallen International Expert Consensus [21]. Intrinsic subtypes were approximated by immunohistochemistry according to international guidelines [22]. Causes of death were obtained from autopsy reports, death certificates and from the national cancer registry (Statistics Finland, Helsinki, Finland).

### Tissue materials

Tissue materials were prepared according to standard histology practice, i.e. fixed in buffered formalin (pH 7.0) and embedded into paraffin blocks. Tissue micro arrays (TMAs) were prepared using the representative tumor area of each patient. The TMAs included two tissue cores with diameters 0.6 mm (cohort I) or 1 mm (cohort II) from each tumor.

### IHC methods

Immunohistochemistry was performed on sections of TMAs cut at 3 μm. Immunohistochemical stainings for Securin, Separase, Cdc20, Pttg1IP, SA2 subunit of Cohesin and CyclinB1 were performed as previously described ([9, 13, 14, 16, 17], Additional file 1). IHC for detecting Cdk1 was performed on an automated immunostaining platform Discovery XT (Roche Diagnostics/Ventana Medical Systems, Tucson, AZ). Deparaffinization, epitope retrieval, and antibody incubation were performed before detection with OmniMap DAB Detection Kit (Roche/Ventana). IHC for Ki-67, estrogen (ER) and progesterone (PR) receptors and HER2, and HER2-amplification with in situ hybridization (ISH) followed standard protocols.

### Interpretation of IHC

Immunoexpressions for Securin, Separase, Cdc20, Pttg1IP, SA2, Cdk1 and CyclinB1 were observed as combinations of nuclear and cytoplasmic staining and registered as average fractions (%) of positively staining cancer cells [13, 14, 16, 23]. In each case, the number of immunopositive cells was calculated in sets of one hundred cancer cells (minimum 100 and maximum 3 × 100 cancer cells evaluated) and registered as an average fraction (%) of immunopositivity for each patient. Interpretations for IHC of Ki-67, ER, PR, and IHC and ISH for HER2 followed previous literature and generally accepted international guidelines [22, 24]. All IHC interpretations were performed by experienced histopathologists (HR, PK).

### Statistical analysis

The cutpoints for immunoexpressions of the studied biomarkers were set based on previous literature, histopathological observations and statistical analyses involving the mean, median and univariate prognostic values of each parameter [9, 13, 14, 16, 17]. In prognostic analyses, Kaplan-Meier estimates were performed to demonstrate the cumulative percentages of breast cancer specific mortality. Cox’s proportional hazard models were used to test associations between disease outcome, biomarker expressions and clinical prognostic features, i.e. tumor size, axillary lymph node status, histological and intrinsic classifications and histological grade. The risk of breast cancer death associated with the studied proteins and the routine prognosticators was quantitated as hazard ratio (HR) with 95% confidence interval (CI). *P*-values < 0.05 were considered statistically significant. The computations were performed with SAS for Windows, Version 9.3 (I-III) and 9.4 (IV) (SAS Institute, Cary, NC, USA). Kaplan-Meier survival plots were generated using R 2.15.0.

## Results

In IHC of the invasive breast carcinomas, Securin was detected as predominantly nuclear but occasionally, showed both nuclear and/or cytoplasmic immunoreaction. Separase showed two distinct and apparently mutually excluding expression patterns observed in the nucleus or in the cytoplasm of cancer cells. Nuclear immunoexpression was observed for Cdc20, SA2, Cdk1 and CyclinB1 whereas Pttg1IP showed cytoplasmic expression only. Table 2 summarizes the fractions of immunopositive breast carcinomas among all breast cancer subtypes (cohort I) and in subgroups divided according to tumor size, nodal status, histological grade and survival.

**Table 2:** Fraction (%) of carcinomas immunopositive for the studied proteins in the whole material and in subgroups

	n	All	N-	N+	T1	T2–3	low grade	high grade	alive	DOD
Securin	604	34	28	52	19	36	28	52	19	42
Separase	401	31	18	32	15	19	17	57	18	34
Cdc20	387	5	3	7	3	6	4	11	4	8
Pttg1IP	420	56	59	53	65	51	66	28	60	51
SA2	470	28	32	22	29	26	28	26	33	19
Cdk1	384	23	9	24	8	24	12	26	6	26
CyclinB1	455	57	53	61	49	60	55	66	51	67

*N*- node-negative, *N+* node-positive, *T1* tumor size < 2 cm, *T2–3* tumor size ≥2 cm, low grade = grades 1–2, *high grade* grade 3, *DOD* dead of disease

The prognostic impacts of the studied regulators of metaphase-anaphase transition were first analyzed for all breast cancer subtypes (cohort I, *n* = 781) with Cox’s proportional hazard model. Among all studied biomarkers, statistically significant prognostic value in univariate analyses was observed for Securin (HR 2.1, *p* < 0.0001, CI 1.6–2.8), nuclear Separase (HR 2.0, *p* < 0.0004, CI 1.4–3.0), Cdk1 (HR 2.5, *p* < 0.0001, CI 1.7–3.6) and CyclinB1 (HR 2.0, *p* = 0.04, CI 1.0–2.0).

**Fig. 1:**
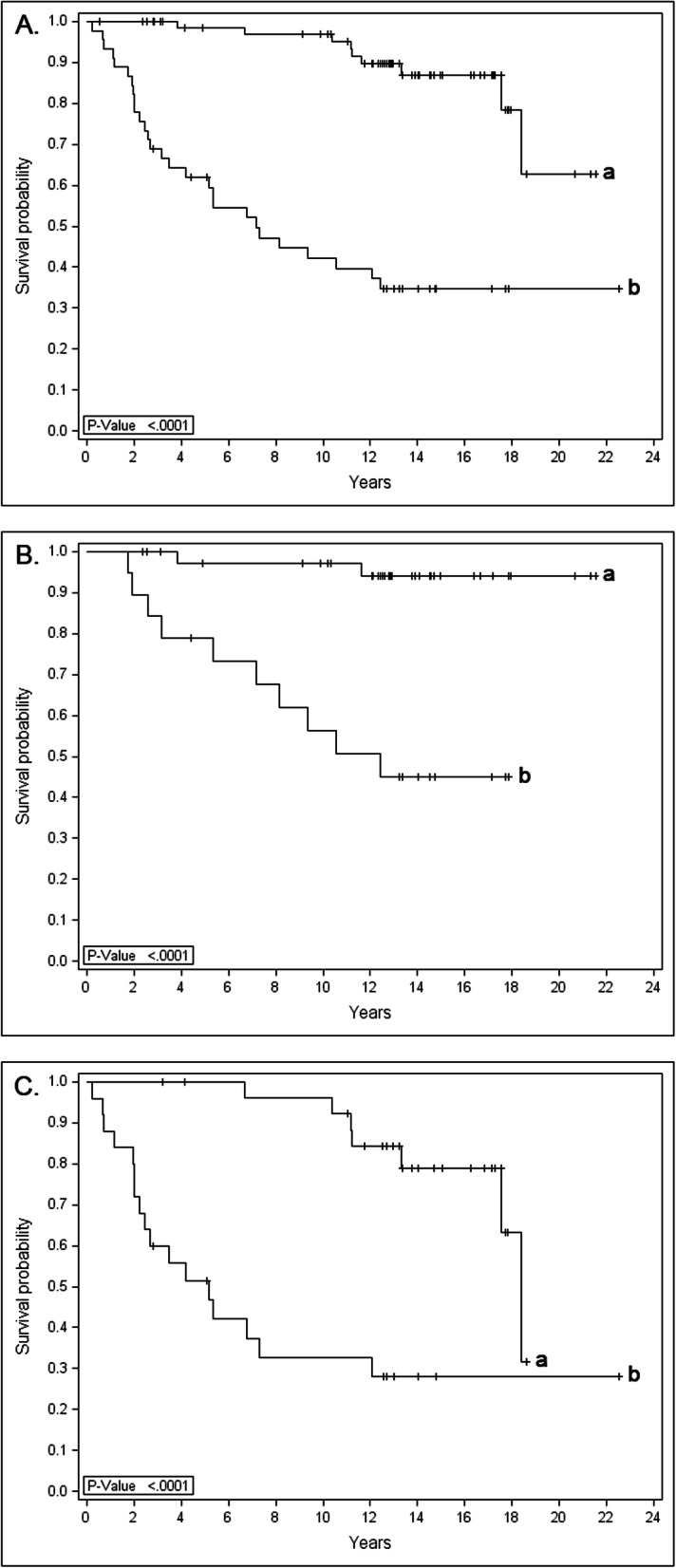
Kaplan-Meier curves show the survival difference between favorable (curve a: low immunoexpressions for Securin, Separase and Cdk1) vs unfavorable (curve b: high expressions of Securin, Separase and Cdk1) outcome of the prognostic model for all breast carcinomas (**a**) and for subgroups with N+ (**b**) and N- (**c**) disease (cohort I, *n* = 781)

In the next phase, these biomarkers were further tested in combinations in order to assemble a prognostic model producing the most significant prognostic impact among all breast cancer subtypes. The optimal model for detecting favorable outcome of disease was determined as the combination of low expressions for Securin (< 10% of cancer cells), Separase (< 1% of cancer cells) and Cdk1 (< 10% of cancer cells). This model was a significant indicator of survival of disease while the opposite detected patients in risk of breast cancer death (HR 8.4, *p* < 0.0001). The Kaplan-Meier curves demonstrate the survival difference among all patients and in subgroups with N+ and N- disease (Fig. 1). Concluding from the survival analyses, favorable outcome of the model indicated that the majority (> 75%) of patients were alive 18.4 years after primary diagnosis while unfavorable outcome of the model suggested that one quarter (25%) of the patients were already dead of breast cancer after 2.5 years of diagnosis. Among the subgroup of N- patients, no cancer-related deaths were observed among patients exhibiting favorable outcome of the model. Instead, the unfavorable outcome suggested cancer mortality for every fourth patient within 5.3 years from diagnosis. Correspondingly, the majority of N+ patients with favorable and unfavorable outcome of the model were alive after 17.6 and 2.0 years from the primary diagnosis, respectively.

Finally, in multivariable analyses of all breast cancer subtypes (Table 3), the designed model was compared with the established clinical prognosticators of breast cancer, i.e. tumor size, nodal status, intrinsic classification and histological grade for superior prognostic power in predicting the risk of breast cancer mortality. In the whole material, significant prognostic impact was observed for axillary lymph node status along with the designed model. Independent prognostic value was detected for the model also among N+ and N- patients. Tumor size (diameter < 2 cm vs ≥2 cm), intrinsic classification or histological grade (1–2 vs 3) were not detected with independent prognostic value in any of the performed analyses.

**Table 3:** Multivariate analyses involving the prognostic model^a^ with nodal status, tumor size, intrinsic classification and histological grade

	HR^b^	p	CI
**All patients** (*n* = 781)			
Model	8.4	< 0.0001	3.4–20.7
Nodal status	4.3	< 0.0001	2.6–7.0
Tumor size		ns.	
Tumor grade		ns.	
Intrinsic classification		ns.	
**N+ patients** (*n* = 350)			
Model	6.5	0.0003	2.3–17.9
Tumor size		ns.	
Tumor grade		ns.	
Intrinsic classification		ns.	
**N- patients** (*n* = 431)			
Model	19.5	0.006	2.3–163.8
Tumor size		ns.	
Tumor grade		ns.	
Intrinsic classification		ns.	

(≥1% of cancer cells) and Cdk1 (≥10% of cancer cells)

^a^High expression for Securin (≥10% of cancer cells), Separase

^b^The hazard ratio of breast cancer death

*ns* not statistically significant

As the cohort I contained a relatively old patient material, a more recent patient material (cohort II) was collected to evaluate the prognostic impact of the regulators of metaphase-anaphase transition in luminal (*n* = 208) and triple-negative breast carcinomas (*n* = 146). As the result of luminal breast carcinomas, Securin (HR 1.1, *p* = 0.02, CI 1.0–1.2) and nuclear Separase (HR 5.7, *p* = 0.002, CI 1.9–17.2) – but not Cdk1 remained the most powerful predictors of cancer mortality, along with nodal status (HR 4.9, *p* = 0.003, CI 1.7–13.7). Also as combined into a model, Securin and Separase showed significant prognostic impact (*p* = 0.0006). Similar trends for the prognostic model were observed also in separate analyses of N+ (*n* = 42) and N- (*n* = 166) patients although the associations were not statistically significant in the small patient groups.

In TNBC (*n* = 146), no statistically significant prognostic value could be detected for any of the immunohistochemically studied biomarkers or clinicopathological features.

## Discussion

In the present study, prognostic models involving regulators of the metaphase-anaphase transition of the cell cycle were introduced for predicting survival of breast cancer patients. The model was assembled based on data from a total of 1135 breast cancer patients with complete clinical information and up to 22-year follow-up.

The results show that combining high immunoexpressions for Securin, Separase and Cdk1 comprises a promising prognostic model indicating 8.4-fold increased risk of breast cancer death (*p* < 0.0001). In luminal breast carcinomas, the combination of Securin and Separase resulted in independent prognostic impact. In all analyses, the prognostic value of the combination of Securin and Separase with or without Cdk1 outperformed the impact of tumor size and histological grade whereas axillary lymph node status remained a strong and independent prognosticator in all analyses. At highest, the introduced prognostic model predicted 19.5-fold increased risk of breast cancer death among N- (*p* = 0.006) and 6.5-fold increased risk of mortality among N+ breast carcinomas (*p* = 0.0003). This suggests that the model may provide additional prognostic information to nodal status in treatment decisions of breast cancer patients. Among N- disease, the model may provide information to identify patients with aggressive course of disease, potentially in need of adjuvant treatments. In N+, the model could provide evidence for withholding chemotherapy from patients with favorable outcome.

TNBC is an aggressive subtype of breast cancer resulting in high mortality. It is also a therapeutically challenging subgroup as the lack of estrogen and progesterone receptors limits the treatment options available. The ongoing intense research has not yet revealed promising novel prognostic biomarkers or treatment targets for TNBC [2]. The present study did not reveal a prognostic impact for the routine clinicopathological parameters or for the studied biomarkers in TNBC. This may be due to the small cohort size as well as the heterogeneous nature of TNBC comprising several molecular subtypes [2].

The value of the introduced model lies in the pivotal role of the studied biomarkers in cancer progression. Loss of control of the cell cycle is a hallmark of malignancy resulting in aneuploidy and genomic instability [25]. Cell cycle checkpoints, including the mitotic checkpoint SAC, are the major cell cycle control mechanism and specifically deregulated in cancer cells. The presently studied regulators of metaphase-anaphase transition are involved in ensuring the fidelity of chromosome segregation. In previous literature, the studied biomarkers have been shown with prognostic impact in breast carcinoma as well as in other malignancies [14, 16, 17, 26–33]. In addition, numerous strategies have been proposed for the design of cell cycle-selective therapies in cancer, including targeting the metaphase-anaphase transition [8]. In all, the introduced model appears to identify biological drivers that could add to the conventional histopathological evaluation on the proliferative behavior of the tumor.

The reliability of the introduced prognostic model is increased by repeated statistical analysis of the studied biomarkers. The first approach applied for all breast cancer subtypes (cohort I) was based on testing combinations of biomarkers for their independent prognostic impact in comparison to each other, the clinical prognostic features and disease survival. In the second approach applied for analysis luminal and triple-negative breast carcinomas (cohort II), the optimal combination of biomarkers was extracted together with the clinical prognostic markers in sequential multivariate analyses of breast cancer-specific survival. Each approach was independently applied on separate patient materials originating from two Finnish breast cancer centers. As a conclusion, irrespective of the patient material or statistical procedure, the combination of Securin and Separase showed the most significant prognostic impact among all breast carcinomas and among luminal breast cancer.

**Fig. 2:**
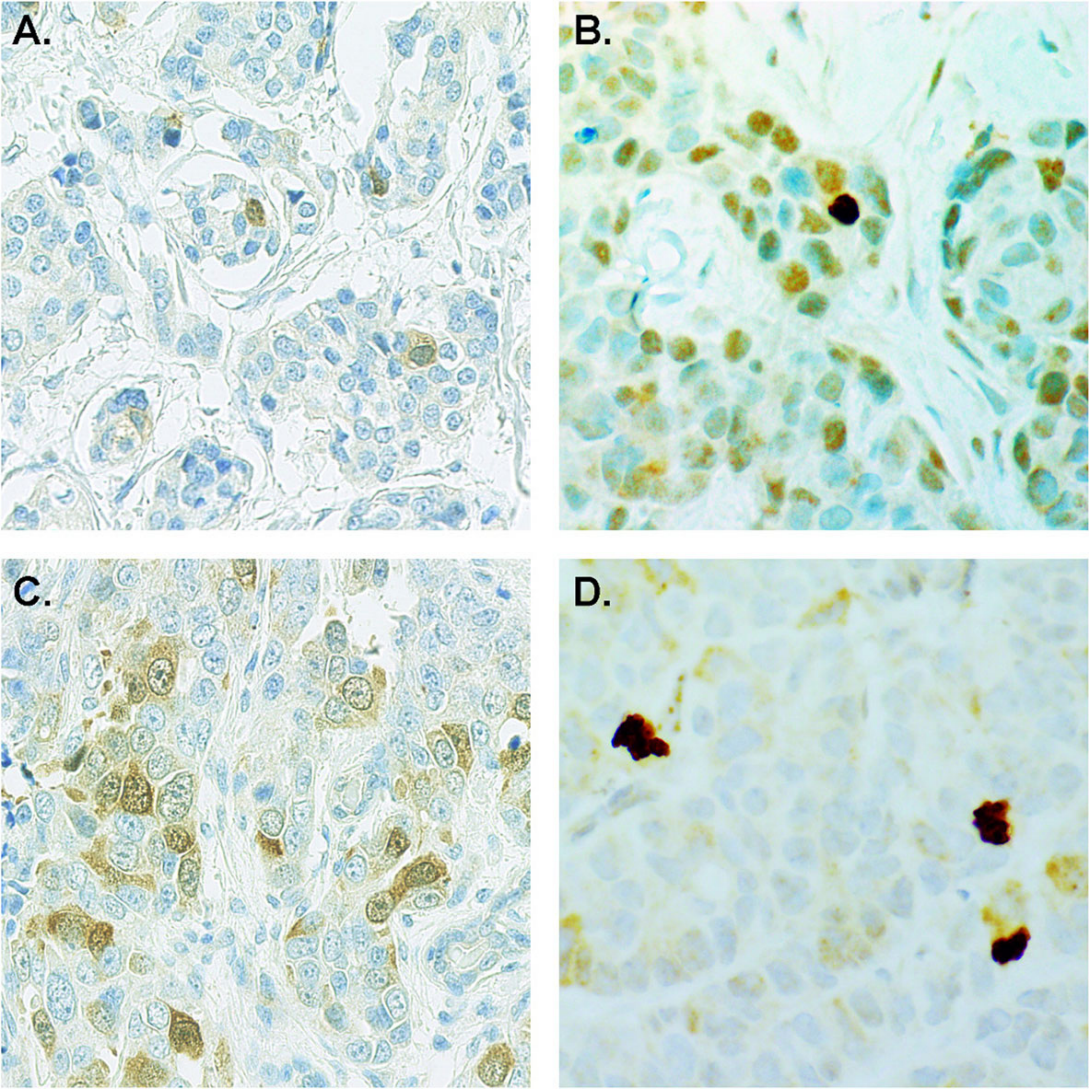
The combination of immunoexpressions for Securin and Separase indicate favorable (Securin **a** and Separase **b**) vs unfavorable (Securin **c** and Separase **d**) outcome of breast cancer

From the practical point of view, immunohistochemical detection of Securin and Separase comprises a biology driven, cost-effective and reliable prognostic method (Fig. 2). By comparison, Ki-67 - the established proliferation marker of cancer diagnostics - has been criticized for high variability across individual pathologist and institutions, as well as for poor prognostic value [34, 35]. In the literature, numerous prognostic models have been introduced for breast cancer but only a few of them have shown impact exceeding that of the routine clinical prognosticators [36–38].

## Conclusions

In our results from a total of 1135 breast cancer patients with complete clinical information and up to 22-year follow-up, high immunoexpression for the combination of Securin and Separase comprises a powerful prognostic tool to identify patients in risk of breast cancer death. In our scenario, the proposed model may facilitate personalized clinical decision suggesting less aggressive therapy for patients associated with low risk of mortality and indicating benefits from adjuvant therapy for a subgroup of patients with aggressive disease. Despite the accumulating data on multiparametric prognostic models, the clinical judgement remains the key determinant on selecting between the different treatment schemes.

## Supplementary information

**Additional file 1.** Details of IHC for detecting Securin, Separase, Cdc20, Pttg1IP, SA2, Cdk1 and CyclinB1 and routine clinical biomarkers of breast cancer ER, PR, Ki-67 and HER2. The datasheet contains the details of the immunohistochemical methods used in this study.

## Data Availability

The data that support the findings of this study are available from Auria Biobank (www.auriabiobank.fi), Turku University Hospital, Turku, and Biobank of Central Finland, Jyväskylä, Finland but restrictions apply to the availability of these data, which were used under license for the current study, and so are not publicly available. Data are however available from the authors upon reasonable request and with permission of the Biobanks stated above.

## Abbreviations

APC/C: Anaphase-Promoting Complex / Cyclosome
Cdc20: Cell division cycle 20
Cdk1: Cyclin-Dependent Kinase protein 1
CI: Confidence interval
CyclinB1: G2/mitotic-specific cyclin-B1
ER: Estrogen receptor
ESPL1: Extra Spindle Pole Bodies Like protein 1
HER2: Human epidermal growth factor 2
HR: Hazard ratio
IHC: Immunohistochemistry
ISH: In situ hybridization
N +: Lymph node positive
N-: Lymph node negative
PBF: Pituitary tumor-transforming gene 1 binding factor
PR: Progesterone receptor
Pttg1: Pituitary tumor-transforming gene 1
Pttg1IP: Pituitary tumor-transforming gene 1 interacting protein
SA2: Stromal antigen 2
SAC: Spindle Assembly Checkpoint
TMA: Tissue microarray
TNBC: Triple negative breast cancer
